# TWEAK Affects Keratinocyte G2/M Growth Arrest and Induces Apoptosis through the Translocation of the AIF Protein to the Nucleus

**DOI:** 10.1371/journal.pone.0033609

**Published:** 2012-03-16

**Authors:** Sanaa Sabour Alaoui, Valérie Dessirier, Elisabeth de Araujo, Vassilia-Ismini Alexaki, Vassiliki Pelekanou, Mustapha Lkhider, Efstathios N. Stathopoulos, Elias Castanas, Martine Bagot, Armand Bensussan, Andreas Tsapis

**Affiliations:** 1 Inserm, U976, Paris, France; 2 Université Paris Diderot, Paris, France; 3 Department of Biology, University of Chouaib Doukkali, El Jadida, Morocco; 4 Laboratory of Experimental Endocrinology, School of Medicine, University of Crete, Heraklion, Greece; 5 Laboratory of Pathology, School of Medicine, University of Crete, Heraklion, Greece; 6 Service de Dermatologie, Hôpital Saint Louis, Paris, France; The Moffitt Cancer Center & Research Institute, United States of America

## Abstract

The soluble TNF-like weak inducer of apoptosis (TWEAK, TNFSF12) binds to the fibroblast growth factor-inducible 14 receptor (FN14, TNFRSF12A) on the cell membrane and induces multiple biological responses, such as proliferation, migration, differentiation, angiogenesis and apoptosis. Previous reports show that TWEAK, which does not contain a death domain in its cytoplasmic tail, induces the apoptosis of tumor cell lines through the induction of TNFα secretion. TWEAK induces apoptosis in human keratinocytes. Our experiments clearly demonstrate that TWEAK does not induce the secretion of TNFα or TRAIL proteins. The use of specific inhibitors and the absence of procaspase-3 cleavage suggest that the apoptosis of keratinocytes follows a caspase- and cathepsin B-independent pathway. Further investigation showed that TWEAK induces a decrease in the mitochondrial membrane potential of keratinocytes. Confocal microscopy showed that TWEAK induces the cleavage and the translocation of apoptosis inducing factor (AIF) from the mitochondria to the nucleus, thus initiating caspase-independent apoptosis. Moreover, TWEAK induces FOXO3 and GADD45 expression, cdc2 phosphorylation and cdc2 and cyclinB1 degradation, resulting in the arrest of cell growth at the G2/M phase. Finally, we report that TWEAK and FN14 are normally expressed in the basal layer of the physiological epidermis and are greatly enhanced in benign (psoriasis) and malignant (squamous cell carcinoma) skin pathologies that are characterized by an inflammatory component. TWEAK might play an essential role in skin homeostasis and pathology.

## Introduction

The TNF-like WEAK inducer of apoptosis (TWEAK) is a member of the TNF ligand superfamily (TNFSF12) that was originally described as a weak inducer of apoptosis in the IFNγ-treated HT-29 colorectal adenocarcinoma cell line [1]. TWEAK is a type II transmembrane protein that can be cleaved to a smaller biologically active soluble form [1], [2], and it binds with high affinity to the fibroblast growth factor-inducible 14 protein (FN14) [3]. Results to date indicate that TWEAK homotrimers do not bind to any other known TNFRSF member and that other known TNFSF homotrimers do not bind FN14 [4]. FN14 (TNFRSF12A) is distantly related to the TNF receptor (TNFR) superfamily member TNFRSF12A, and it contains only one cysteine-rich domain in its extracellular region and a TNFR-associated factor (TRAF) binding domain, but no death domain (DD) in its cytoplasmic tail [3]. The presence of a death domain in a TNFR-type receptor is generally considered indicative of its ability to induce apoptosis. Upon binding of its cognate ligand, a DD-containing TNFR recruits pro-apoptotic adaptive proteins and initiates the extrinsic pathway of apoptosis through caspase activation. However, a number of TNFR family members that lack canonical death domains also trigger cell death in an *in vitro* setting [5], [6], [7], [8], [9], [10].

Soluble TWEAK induces a variety of biological responses, including cell growth and proliferation [11], angiogenesis [12], [13], osteoclastogenesis [14], migration [15] and apoptosis [1], [16], [17]. FN14 is reported as the unique signaling-competent receptor that mediates TWEAK activity in all cell types [4].

The precise signaling pathways that lead to TWEAK-induced cell death are not well understood but appear to involve multiple context-dependent mechanisms, including TNF-dependent apoptosis [9], TNF-independent caspase-dependent apoptosis, caspase-independent death with features of both apoptosis and necrosis, and cathepsin B-dependent necrosis [10], [11], [16], [18]. Previous reports indicate that depending on the cell type, the TWEAK/FN14 interaction activates two main pathways: the NF-κB (canonical and alternative) signaling pathway [11], [18], [19], [20] and the MAPKinase (MAPK), JNK (HUVECs) [15], p38 and Erk (HEK293, MC3T3-E1) [15], [21], [22] signaling pathways. However, recent studies of Kym-1 [9], HSC-3 [23] and other tumor cell lines [24], suggest a unique caspase-dependent apoptosis mechanism that results from an increase in TNFα secretion and its binding to the TNFR1 receptor. These diverse and distinct TWEAK activities are reviewed by Winkles, 2008 [25].

In a recent report describing the soluble factors implicated in keratinocyte destruction during the onset of Lyell's syndrome, we have determined the presence of TWEAK in Lyell blister fluids and have shown that TWEAK induces the apoptosis of keratinocytes *in vitro* [26]. Because TNFα does not induce apoptosis but arrests keratinocyte cell growth [27], we investigated the mechanism of TWEAK-induced apoptosis in keratinocytes using two different human epidermal cells, the HaCaT cell line and normal human foreskin keratinocytes (NHK). Our data reveal that TWEAK alone can induce normal human keratinocyte cell death in a caspase- and cathepsin B-independent manner. In addition, through a TNFα- independent pathway, TWEAK induces a decrease in mitochondrial membrane potential and the cleavage and translocation of AIF (apoptosis inducing factor) from mitochondria to the nucleus. TWEAK also induces cdc2/cyclinB1 complex degradation and cell growth arrest at the G2/M phase. Finally, we report the presence of TWEAK and FN14 in normal human epidermis and skin appendices, where they exhibit a discrete expression, whereas their increased expression in cases of malignant and benign lesions of human epidermis suggests a role for TWEAK in inflammatory skin pathologies.

## Results

### HaCaT cells and normal human keratinocytes (NHK) bind TWEAK through the FN14 receptor

**Figure 1:**
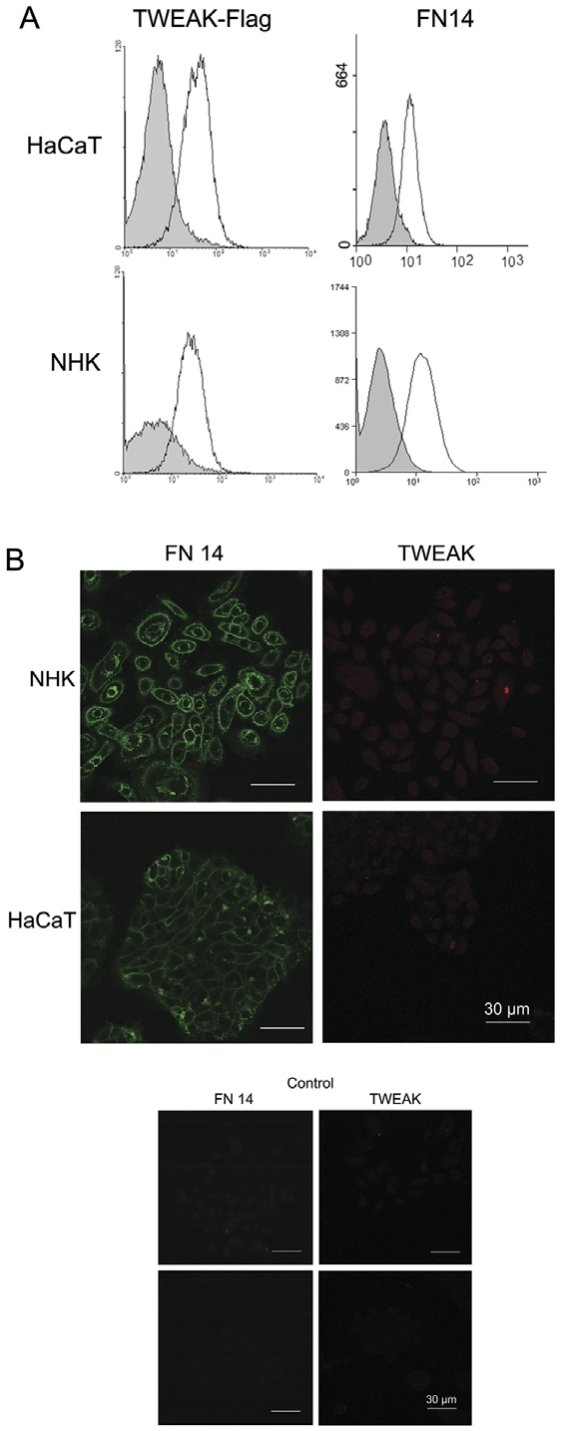
FN14 is present on the cell surface and binds TWEAK. A. The HaCaT cell line and normal human keratinocytes (NHK) were incubated in the presence of FLAG-tagged TWEAK and anti-human FN14 FITC-conjugated mAb. Control experiments were performed using cells incubated with buffer alone or in the presence of irrelevant mouse IgG FITC-conjugated antibodies. TWEAK binding was detected by the addition of the monoclonal M2 anti-FLAG antibody followed by the addition of a PE-conjugated anti-mouse IgG goat antibody. The grey area represents the mock control experiments, and the continuous line represents flagged-TWEAK, and anti-FN14 bound molecules. B. HaCaT cells and NHK were cytospun onto glass slides, were fixed and were stained using specific anti-FN14 FITC-conjugated and anti-TWEAK Texas red-conjugated mAbs. The slides were mounted and photographed as described in the Materials and Methods section.

To determine if TWEAK affects keratinocytes, we first examined the binding of TWEAK to HaCaT cells and NHK. Flow cytometry analysis using a FLAG-tagged human recombinant TWEAK showed that TWEAK binds both HaCaT and NHK, suggesting the expression of its receptor on the cell surface. The use of an anti-human FN14 mAb demonstrated that FN14 is present on the surface of both HaCaT cells and NHK (Figure 1A). Using immunofluorescence, we detected the presence of FN14 on the cell surface and in the Golgi apparatus of both HaCaT cells and NHK, and a faint staining for TWEAK was observed in the cytoplasm of these cells (Figure 1B).

### TWEAK induces the apoptosis of HaCaT cells and NHK

**Figure 2:**
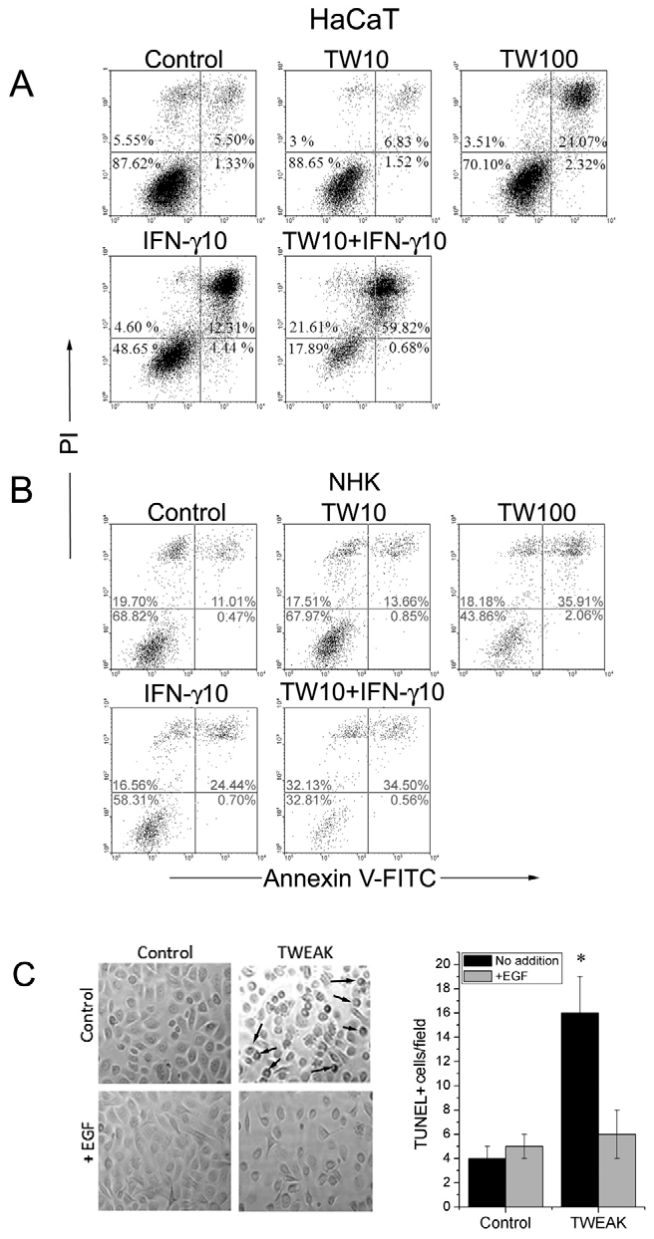
TWEAK induces apoptosis of keratinocytes. TWEAK induces the apoptosis of HaCaT cells (A) and NHKs (B) in a dose-dependent manner. HaCaT cells and NHKs were incubated in culture dishes with increasing amounts of TWEAK (10–100 ng/ml) and IFN-γ (10 ng/ml) for 48 h. The cells (including floaters) were collected and incubated with annexinV-FITC and propidium iodide for flow cytometry analysis. Control experiments were performed by incubating the cells in cell culture medium. The numbers indicate the percentage of cells within the indicated gate. The results shown are representative of three independent experiments. C. Normal human keratinocytes were examined under a microscope after the addition of recombinant TWEAK (100 ng/ml) or/and EGF (50 ng/ml) for 48 h. The characteristic membrane blebbing of the cells is shown by arrows, and quantification was performed using the TUNEL method. *P* values were calculated by comparing the treated cells to their relevant controls and were considered significant when *P*<0.05.

TWEAK induces cell death in a variety of tumor cell lines by increasing cell apoptosis [10], [11], [16], [18]. A previous report showed that TWEAK decreases the viability of HaCaT cells and NHK, and that this decrease is enhanced by the simultaneous addition of the inflammatory cytokine IFNγ [26]. Therefore, we investigated whether the decreased growth that is observed in HaCaT cells and primary keratinocytes relies on increased apoptosis. The cells were incubated with variable concentrations of TWEAK and/or IFNγ for 48 h. We observed a slight increase in the number of HaCaT and NHK annexin V-single positive cells, and the number of annexinV/PI double positive cells increased in a dose-dependent manner. The incubation of cells with IFNγ doubled the population of annexinV single positive cells, and the number of annexinV/PI double positive late apoptotic cells was markedly increased (8-fold in HaCaT and 2-fold in NHK cells, respectively) compared to controls. An additive maximal apoptotic effect was observed following the simultaneous addition of low doses (10 ng/ml) of TWEAK and IFNγ. The results are shown in Figure 2A for HaCaT cell line and in Figure 2B for normal human keratinocytes (NHK).

These results were confirmed by the visualization of membrane blebbing in the NHKs after treatment with TWEAK and using a terminal deoxynucleotidyl transferase dUTP nick end labeling (TUNEL) assay. Because our culture medium contains no recombinant EGF and a low concentration of bovine pituitary extracts (6 µg/ml), we also examined apoptosis after the simultaneous addition of 50 ng/ml EGF and 100 ng/ml TWEAK. The results obtained clearly demonstrate that the addition of EGF reverses the apoptosis that is induced by the addition of TWEAK (Figure 2C).

### TWEAK and FN14 are expressed in normal epidermis and appendices, and their expression is increased in inflammatory skin lesions

**Figure 3:**
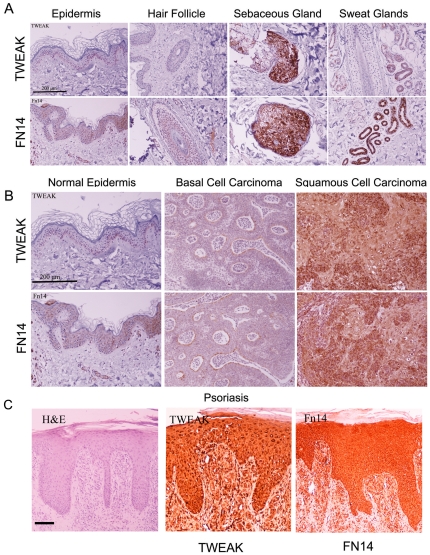
FN14 and TWEAK are detected in normal and pathological skin. Representative sections of normal epidermis, hair follicles, and sebaceous and sweat glands (A), sections of epidermis from patients suffering from basal cell carcinoma, squamous cell carcinoma (B) and psoriasis (C) were stained for FN14 and TWEAK. Bar represents 200 µm.

Using immunohistochemical staining, we investigated whether TWEAK and/or its receptor FN14 are present in normal epidermal structures and skin appendices (hair follicle, sebaceous sweat glands). In normal skin, TWEAK is expressed only at the basal layer of normal epidermis; however, its receptor FN14 is present in the basal and in the upper (spinosal and granulosal) layers, albeit in a heterogeneous manner. Hair follicles stain weakly for FN14, whereas sebaceous glands and especially sweat glands stain strongly for FN14 and TWEAK (Figure 3A).

Finally, we examined the expression of TWEAK and FN14 in inflammatory and non-inflammatory skin pathologies. In basal cell carcinomas TWEAK and Fn14 are highly expressed only in palisading cells (Figure 3B). In contrast, TWEAK and FN14 are highly expressed in squamous cell carcinoma, a tumor that is characterized by an intense inflammatory component (Figure 3B). In psoriasis (Figure 3C), a benign skin lesion that is characterized by intense inflammation, the keratinocytes heavily express TWEAK and FN14.

### Death of keratinocytes observed following the addition of TWEAK is not due to the secretion of TNFα, TRAIL or another death-inducing TNF ligand

**Figure 4:**
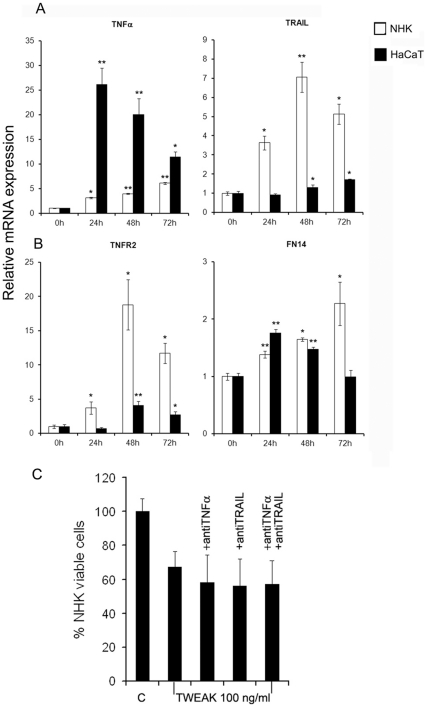
Modification of the expression of death ligands and receptors in keratinocytes following the addition of TWEAK. The relative expression of TNFα and TRAIL (A), TNFR2 and FN14(B) mRNAs in HaCaT cells and NHK compared with the expression of GAPDH mRNA. The results were normalized to the expression of control non-treated cells, which was given a value of 1. TWEAK was added at a concentration of 100 ng/ml. C. NHK were pre-incubated with neutralizing anti-TNFα (100 ng/ml) and anti-TRAIL (100 ng/ml) monoclonal antibodies before the addition of 100 ng/ml TWEAK. Cell viability in the presence and absence of neutralizing antibodies was measured 48 h later and is expressed as a percentage of the viability of control cells.

Previous reports have shown that the addition of TWEAK to several tumor cell lines results in an increased production of TNFα, which is responsible for the observed death phenomenon [25]. In this study, we assayed by RT-PCR whether a number of TNFSF ligands (TRAIL, FasL, LTα, EDA A1) and receptors (TNFR1 and 2, Fas, EDAR) as well as TWEAK's cognitive receptor FN14 are modulated by the addition of TWEAK.. We observed no significant increase in the mRNA expression of FasL, LTα (lymphotoxin α) or EDA A1 ligands or TNFR1, Fas, and EDAR receptors. Addition of TWEAK strongly stimulated the expression of TNFα mRNA in both HaCaT and NHK, and a smaller increase in the expression of TRAIL mRNA was detected (Figure 4A).

The results of secretion of both TNFα and TRAIL by keratinocytes in presence or absence of TWEAK are shown in Table 1. TNFα secretion is very low in both HaCaT and NHK control samples and it is not significantly modified by the addition of TWEAK. TRAIL secretion is increased in both control HaCaT and primary keratinocytes over the time course. However the addition of TWEAK to HaCaT cell line, decreases TRAIL expression as compared to that of control. For primary keratinocytes(NHK) addition of TWEAK does not modify significantly the secretion of TRAIL. In conclusion, the addition of TWEAK does not increase the secretion of either TNFα and/or TRAIL by both HaCaT cell line and primary foreskin keratinocytes as compared to that of control samples. This was confirmed using flow cytometry, which demonstrated the absence of TNFα and TRAIL protein staining in the cytoplasm of both cells following the addition of TWEAK. Finally, the incubation of NHK with neutralizing anti-TNFα and anti-TRAIL monoclonal antibodies in the presence or absence of TWEAK did not alter cell viability (Figure 4C). These results suggest that under our experimental conditions, there is no effective increase translation and/or secretion of TNFα and TRAIL proteins upon addition of TWEAK, and these pro-apoptotic TNFα and TRAIL ligands are not implicated in the apoptosis of keratinocytes that is observed following the addition of TWEAK.

**Table 1:** Secretion of TNFα and TRAIL by keratinocytes upon addition of 100 ng/ml of TWEAK.

	TNFα pg/ml			TRAIL pg/ml		
	24 h	48 h	72 h	24 h	48 h	72 h
**HaCaT control**	16±4	3±1	20±5	186±19	346±17	686±16
**HaCaT+TWEAK**	11±5	0	12±4	78±1	243±123	112±92
**NHK control**	25±1.5	32±0.5	N.D.	30±20	350±37	N.D.
**NHK+TWEAK**	36±2	63±19	N.D.	65±8	375±118	N.D.

We also observed a significant increase in TNFR2 and FN14 mRNA expression (Figure 4B).

### TWEAK induces apoptosis through a caspase- and cathepsin B-independent pathway

**Figure 5:**
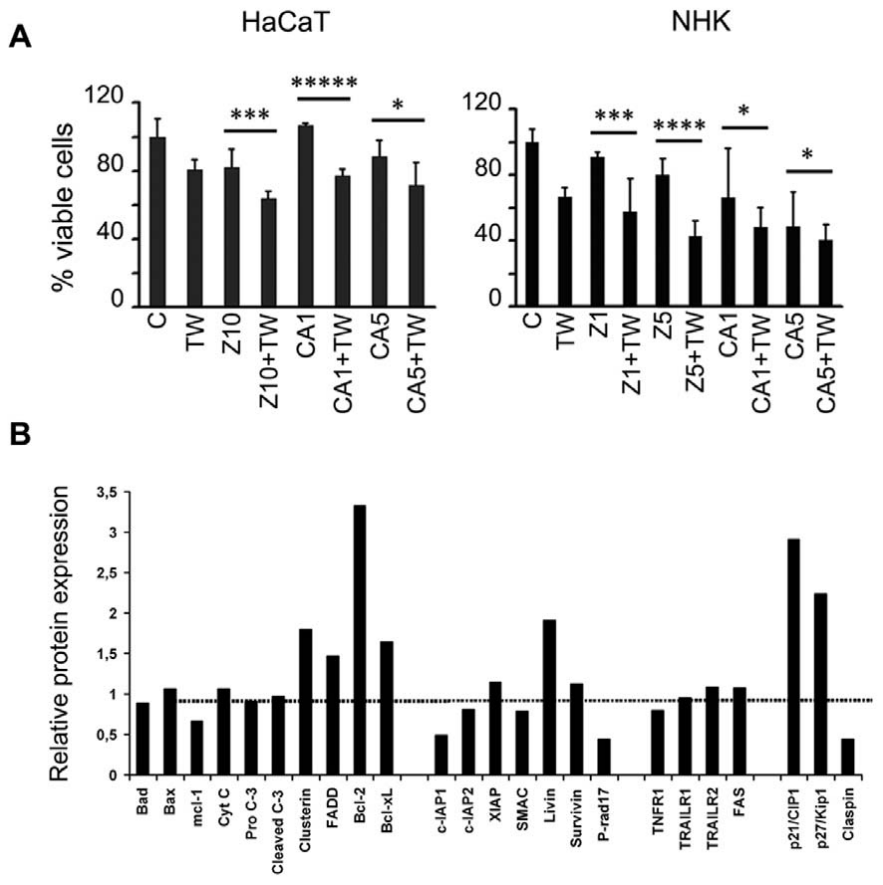
Apoptosis of keratinocytes is caspase- and cathepsin B-independent. A. HaCaT cells (5.10^3^ cells/well) or NHK (10.10^3^ cells/well) were incubated with the indicated concentrations of caspase (zVAD-fmk) or cathepsin B (CA-074) inhibitors for 48 h (HaCaT) or 72 h (NHK) in the presence or absence of 100 ng/ml recombinant TWEAK. The cell viability was assessed using an MTT assay. The results are expressed as a percentage, with the proliferation of unstimulated cells set at 100% the (mean of triplicates ± SEM). The data are representative of three independent experiments. TWEAK and the inhibitors were not added to the control cells; however, DMSO at a concentration equal to that in the inhibitor solutions was added. Z = Zvad-fmk; C = CA-076; 1, 5 or 10 corresponds to 1, 5 or 10 µM, respectively; TW = TWEAK). *P* values were calculated by comparing the treated cells to the relevant controls, and were considered significant when *P*<0.05. B. NHK were treated with 100 ng/ml TWEAK for 24 h. The cells were lysed, and the expression of several pro- and anti-apoptotic proteins was measured as described in the Materials and Methods section. The relative expression of these proteins compared with that of cells not treated with TWEAK is presented. The controls are considered as 100% (dotted line).

Several studies report that depending on the cell type, TWEAK-induced cell death involves multiple mechanisms, including the activation of cathepsin B and caspases [11], [18]. We used the general caspase inhibitor zVAD-fmk and the cathepsin B inhibitor CA-074 to assess the role of caspases and cathepsin B in NHK and HaCaT cell death, respectively. Before assaying for cell growth, the cells were pre-treated with different concentrations (1, 5 and 10 µM) of either zVAD-fmk or CA-074 and were incubated at 37°C for 2 days (HaCaT) or 3 days (NHK) in the presence of 100 ng/ml TWEAK. Because the inhibitors were dissolved in dimethylsulfoxide (DMSO), vehicle concentrations were used as controls (0.1%, 0.05% and 0.01% DMSO). Our data demonstrate that a concentration of 0.1% DMSO is deleterious to primary keratinocytes (data not shown); therefore, only 1 µM and 5 µM inhibitor concentrations were used for experiments involving NHK. The addition of zVAD-fmk or CA-076 does not restore the decreased cell viability caused by the addition of TWEAK (100 ng/ml) in HaCaT or NHK cells (Figure 5A), suggesting that the mechanisms of TWEAK-induced keratinocyte death are independent of caspase and cathepsin B activities.

We also assayed the expression levels of several pro- and anti-apoptotic proteins using a protein array (described in Materials and Methods) 24 h after the addition of 100 ng/ml TWEAK to NHKs. The results are expressed as the fold-change in expression of the proteins compared to untreated cells (considered as 1) and are presented in Figure 5B. There was no significant modification in the expression of the pro-apoptotic proteins Bad and Bax or cytochrome C. In contrast, the mcl-1 anti-apoptotic protein expression decreased by ∼40%, whereas expression of the anti-apoptotic proteins Bcl-2 and Bcl-xL increased. There was a 50% increase in the expression of pro-apoptotic FADD protein. We also detected a decrease in the expression of c-IAP1, c-IAP2 and SMAC, which are inhibitors of apoptosis, but there was an increase in the expression of livin (another member of the family of IAP proteins) and no increase in XIAP protein expression. In accordance with the results described above (Figure 5A), there was no modification of pro-caspase-3 and cleaved caspase-3 expression, which indicates that caspases are not implicated in apoptosis. Furthermore, as all these pro- and anti- apoptotic proteins (members of bcl2 or IAP families) usually activate the cascade of caspases, we can reasonably suggest that their modulation does not influence the apoptosis phenomenon. No significant change in the expression of TRAILR1, TRAILR2 and FAS, and a small decrease in the expression of TNFR1 were observed. These data, investigating pro-apoptotic and anti-apoptotic proteins expression, combined to that of absence of caspase activation indicate that we have to investigate the caspase-independent pathways of apoptosis to find out the pathway through which keratinocytes commit to cell death upon the addition of TWEAK.

The increase in expression observed for p21/CIP1 (3-fold increase), and p27/Kip1 (2-fold increase) has been associated with the arrest of cell growth at G1 [28] and G2 phases [29]. The decrease(60%) in the expression of claspin, a cell cycle-regulated protein that peaks at the S/G_2_ phase and the expression of which is related to the function of the S phase of the cell cycle [30]. These data may indicate a TWEAK-induced modification of cell cycle regulation.

**Figure 6:**
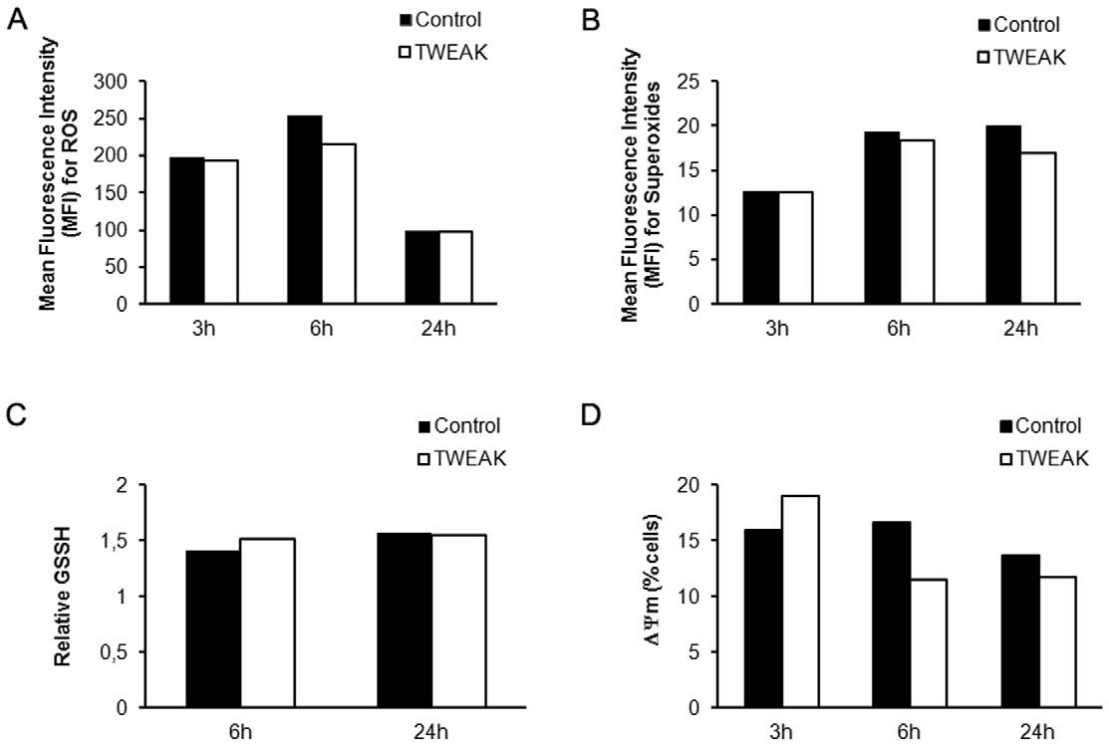
TWEAK decreases the mitochondrial membrane potential of keratinocytes. NHK were treated with TWEAK (100 ng/ml) and ROS (A), and superoxides (B), oxidized glutathione (C) and the mitochondrial membrane potential (C) was measured as indicated in the Materials and Methods section.

We have also determined whether the addition of TWEAK induces a modification of the oxidative status of keratinocytes. In this respect we have assayed the production of ROS (Reactive Oxygen Species), superoxide ions, oxidized glutathione and the modification of mitochondrial membrane potential after incubation of keratinocytes with 100 ng/ml of TWEAK. The results obtained clearly indicate that the addition of TWEAK does not result in the significant production of ROS (Figure 6A), or superoxide ions (Figure 6B), or an increase in oxidized glutathione levels (Figure 6C). On the contrary we have found that TWEAK decreases significantly the mitochondrial membrane potential (Figure 6D) of the keratinocytes, suggesting a mitochondrion –related apoptosis..

### Addition of TWEAK results in the cleavage and the translocation of AIF (apoptosis inducing factor) from the mitochondria to the nucleus

**Figure 7:**
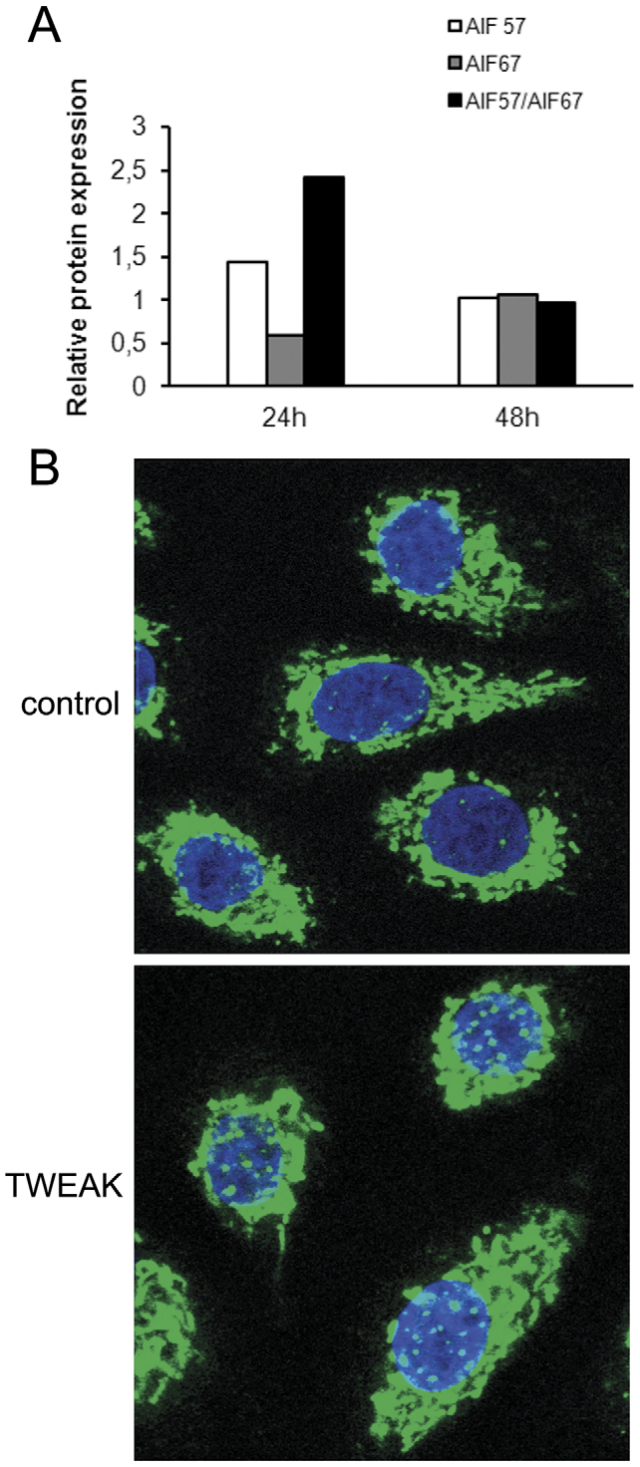
AIF translocates to the nucleus following the addition of TWEAK. A. NHK cells were incubated with TWEAK (100 ng/ml). The cells were lysed at various time intervals and were analyzed by Western blotting with a rabbit polyclonal anti-AIF specific antibody. B. The control cells were left untreated. The cells were detached, placed on glass slides, fixed with paraformaldehyde, permeabilized with Triton X-100 and stained with a rabbit polyclonal anti-AIF antibody. The bands were visualized using donkey anti-rabbit F(ab)′_2_ FITC polyclonal antibody. The nucleus was stained with DAPI.

The data, already obtained, clearly indicate a caspase-independent mechanism of TWEAK action in apoptosis. Therefore, we investigated whether AIF, which is known to induce caspase-independent apoptosis, is implicated in the TWEAK-induced apoptosis of keratinocytes. AIF is an inner mitochondrial membrane protein of 67 kDa, which is cleaved to a 57 kDa species, prior to its release to the cytosol first and its consequent translation to the nucleus in which it initiates chromatin condensation and DNA fragmentation [31]. As shown in Figure 7A, addition of TWEAK decreases the AIF 67 kDa species and induces an increase of mature AIF 57 kDa species. This is transient phenomenon with a peak at 24 h incubation of TWEAK. Following the addition of TWEAK, a partial translocation of AIF to the nucleus occurs (Figure 7B), suggesting that AIF is implicated in the observed apoptosis phenomenon.

### Addition of TWEAK induces keratinocyte G2/M growth arrest due to cdc2 phosphorylation and cdc2 and cyclin B1 degradation

**Figure 8:**
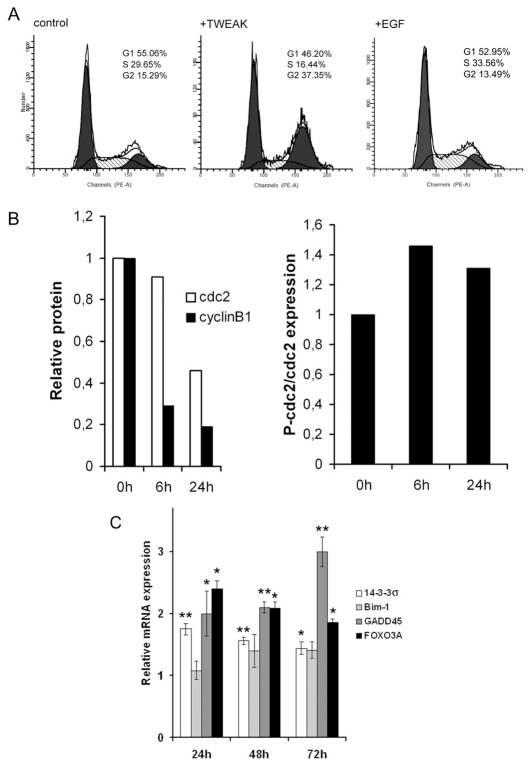
TWEAK induces the cell growth arrest of keratinocytes and the degradation of the cdc2/cyclin B1 complex, and activates FOXO3A, GADD45 and 14-3-3σ. A. NHK cells were treated with TWEAK (100 ng/ml) or TWEAK and EGF (50 ng/ml) for 24 h. The nuclei were isolated, were stained using propidium iodide and the cells were counted using flow cytometry. The results were analyzed using a ModFit program. Untreated cells were used as controls. B. NHK cells were incubated with TWEAK (100 ng/ml). The cells were lysed at various time intervals and were analyzed by Western blotting with anti-cyclin B1, anti-cdc2 and anti-phospho-cdc2 specific antibodies. C. NHK cells were treated with TWEAK (100 ng/ml) for 24, 48 and 72 hours. The cells were lysed, and the RNA was isolated. A quantitative RT-PCR assay using specific primers for FOXO3A, GADD45, Bim-1 and 14-3-3σ mRNAs was used to measure the expression of these genes. GAPDH mRNA expression was used to normalize the results, which are expressed as a fold increment compared to that of the control sample, which was given a value of 1. A statistical analysis (the Student's *t*-test) was performed and the results were considered significant when *P*<0.05.

The modification of p21, p27 and claspin expression following the addition of TWEAK indicates a possible modulation of the cell cycle. To investigate this hypothesis, keratinocytes were treated for 24 hours with 100 ng/ml recombinant TWEAK, and the cell cycle was analyzed and compared to that of untreated keratinocytes. As shown in Figure 8A, TWEAK blocks the keratinocyte cell cycle at the G2/M phase. The simultaneous addition of recombinant EGF and TWEAK reverses the cell cycle growth arrest that is observed upon the addition of TWEAK alone, supporting the data presented in Figure 2C that demonstrates the reversion of the TWEAK apoptotic effect by the addition of EGF.

To further analyze this effect of TWEAK on the cell cycle, we have investigated the fate of cdc2, phosphorylated cdc2 and cyclinB1 proteins, which control the G2/M checkpoint. The results presented in Figure 8B clearly show that the addition of TWEAK significantly decreases the level of cdc2 and cyclinB1 proteins and increases the level of phosphorylated cdc2, suggesting that G2/M growth arrest is due to the degradation of the cdc2/cyclinB complex, as previously described in primary keratinocytes [32].

FOXO transcription factors are known to trigger a variety of cellular processes and especially in our case , apoptosis and cell cycle arrest by up regulating target genes as, p21, p27, Bim-1, FASL, GADD45, 14-3-3σ, depending on the environment of the cell and the stimuli exerted on it [33]. We have already reported that TWEAK does not increase FASL transcription and shown that it increases the expression of p21 and p27 proteins (Figure 5B). We have used qRT-PCR assays to investigate the expression of FOXO3A, GADD45 and 14-3-3σ mRNA, and to better define the pathway implicated in the arrest of cell growth at the G2/M phase. The results presented in Figure 8C show that the addition of TWEAK, does not increase Bim 1 expression (excluding the participation of Bim 1 in the TWEAK-induced apoptosis of keratinocytes) while it increases significantly the expression of FOXO3A, GADD45 and 14-3-3σ mRNAs.We have shown for the first time in our knowledge that the addition of TWEAK increases the transcription of FOXO3A. GADD45 [34], [35], [36] and 14-3-3σ [37] are known to bind to cdc2 inhibiting the kinase activity and induce cell growth arrest at G2/M phase.

## Discussion

The tumor necrosis factor superfamily (TNFSF) consists of ligands that mediate their effect through the engagement of TNF receptor superfamily (TNFRSF) members. Members of the TNFRSF, which contain a specific “death domain” sequence in their cytoplasmic tail, recruit adaptor proteins that trigger caspase activation and result in the activation of the extrinsic pathway of apoptosis. The TNF-like weak inducer of apoptosis (TWEAK, TNFSF12) is a relatively new member of this superfamily [1] that specifically binds to the FN14 receptor [3] and regulates a number of physiological [4], [25] and pathological processes [38], such as growth, differentiation and/or apoptosis. The FN14 receptor does not contain a “death domain” that directly triggers apoptosis. Previous studies suggest that the addition of TWEAK results in an increase in the secretion of TNFα, which binds to TNFR1 and triggers the extrinsic pathway of apoptosis [9], [23], [24]. In this study, we have shown that recombinant FLAG-tagged TWEAK binds its FN14 receptor on the surface of HaCaT cells and NHKs, suggesting that human keratinocytes express cell surface FN14 and may therefore respond to TWEAK stimulation. TWEAK treatment induced the apoptosis of HaCaT cells and NHKs. Because the addition of TNFα results in only cell growth arrest, in these cells [27], we have investigated mechanisms that govern the pro-apoptotic action of TWEAK.

A recent study [39] suggests that TWEAK alone cannot induce the apoptosis of keratinocytes, whereas we have shown that TWEAK induces a 26% and 38% apoptosis in the HaCaT cell line and NHK respectively. The discrepancy in these results is due, in our opinion, to the factors introduced in the keratinocyte culture medium; SFM medium used in the previous study [39] contained 5 ng/ml EGF and 50 µg/ml bovine pituitary extracts, whereas our CnT-57 medium contains only 6 µg/ml of bovine pituitary extracts and no EGF. Following the addition of EGF, we show that the pro-apoptotic effect of TWEAK is abolished, supporting our hypothesis. Moreover, TWEAK induces apoptosis of the HaCaT cell line, which is grown in a medium that does not contain EGF.

Furthermore, we have investigated whether the addition of TWEAK triggers an increase in the expression and secretion of TNFα, as proposed in other reports [9], [23], [24]. We have extended our study to include the death ligands TRAIL, FasL, LTα and EDA-A1 and their corresponding receptor mRNAs. The results confirm that TWEAK induces an increase in TNFα mRNA expression and a small increase in TRAIL mRNA expression. Surprisingly, the increases in the mRNA expression levels do not result in an increase in the amount of TNFα and TRAIL proteins secreted by HaCaT cells and NHK following incubation with TWEAK. Furthermore, the addition of neutralizing antibodies does not restore the viability of the cells. This latter fact excludes TNFα and/or TRAIL as the potential mediators of TWEAK-induced apoptosis. Our results that pan-caspase and cathepsin B inhibitors do not restore the viability of keratinocytes and the observation that caspase-3 is not activated strengthen the suggestion that the TWEAK-induced apoptosis of keratinocytes is caspase- and cathepsin B-independent and that TNFα and TRAIL, which induce caspase activation when bind their corresponding receptors, do not mediate TWEAK-induced apoptosis.

Because it is clear that the apoptosis observed is caspase-independent and that TWEAK induces changes in the mitochondrial potential, we have investigated the implication of the translocation of apoptosis inducing factor (AIF), which is known to induce caspase-independent apoptosis [31], [40] from the mitochondria to the nucleus. We showed that addition of TWEAK results in cleavage of membrane bound AIF, a prerequisite step for the translation of AIF from mitochondria to nucleus [31]. Confocal microscopy demonstrating the translocation of AIF to the nucleus suggests that AIF is responsible for the caspase-independent apoptosis observed in keratinocytes. The AIF activation mechanism that is reported here for the first time may explain previous results demonstrating a caspase-independent apoptosis in different cell lines following the addition of TWEAK [10].

We have shown that TWEAK induces cell growth arrest at G2/M through both the transcription of FOXO3A, GADD45 and 14-3-3σ, and the degradation of the cdc2/cyclinB1 complex. GADD45 inhibits the kinase activity by binding to cdc2 [34], [35], [36] and 14-3-3σ by sequestering cdc2/cyclinB1 complex in the cytosol [37]. Furthermore we have observed the increase of p21 and p27 proteins known to inhibit cdc2 kinase and to induce a G2/M growth arrest [41], [42]. Here, we are in presence of activation of more than one mechanism that could be involved in the G2/M growth arrest observed. Further investigation is necessary to estimate the relative importance of each mechanism implicated in keratinocyte growth arrest.

Because the addition of EGF abolishes the cell growth arrest and apoptosis observed after the addition of TWEAK, this suggests that cell growth arrest and apoptosis are related, with cell growth arrest preceding apoptosis.

The data reported in this paper suggest that TWEAK-induced apoptosis of keratinocytes is caspase-independent and takes place through opening of mitochondrial membrane cleavage and translocation of AIF to the nucleus. Work is in progress in our group to better define the molecular basis of the correlation between G2/M cell growth arrest and the apoptosis observed.

TWEAK and FN14 are present in the normal epidermal structure and skin appendices (hair follicle, sebaceous gland and sweat gland), and an increased expression of TWEAK and FN14 is found in inflammatory benign and malignant skin conditions, such as psoriasis and squamous cell carcinoma. The data suggest that TWEAK and FN14 are involved in skin physiology. Notably, a similar high level of TWEAK and FN14 expression has been reported in renal tubules [43] and in a number of vessels, suggesting that TWEAK is involved in secretory processes. A recent report [26] shows that TWEAK is involved in the death of keratinocytes in toxic epidermal necrolysis. Taken together with the data presented here that demonstrates the direct pro-apoptotic effect of TWEAK in human keratinocytes, this study strengthens our hypothesis that TWEAK plays a role in immune and/or remodeling reactions in healthy and in pathological skin.

## Materials and Methods

### Cells and reagents

The HaCaT cell line is a keratinocyte-derived cell line [44]. HaCaT cells were grown in Dulbecco's modified minimal essential medium (DMEM) supplemented with 10% fetal calf serum (FCS) and 5 mg/ml plasmocin™ (Cayla, Toulouse, France) antibiotic. In the viability assays, DMEM was replaced with RPMI 1640 medium for optical density measurement considerations. Normal human foreskin keratinocytes (NHK) were grown in pre-coated plastic dishes containing Epilife® medium that was supplemented with defined growth supplement (EDGS), according to the manufacturer's instructions. For routine subculture, a synthetic coating mix was used; 96-well plates were prepared using a collagen gel that was reconstituted from an acid soluble rat-tail collagen I solution. All media and supplements were purchased from Invitrogen (Cergy Pontoise, France), with the exception of soluble rat-tail collagen I, which was purchased from BD Biosciences (Le Pont de Claix, France). CnT-57 medium (CellnTec, Bern, Switzerland) was also used for keratinocyte culture, which does not require the flasks to be coated with collagen. The results obtained using the two different media were similar. The cells were maintained at 37°C in an atmosphere containing 5% CO_2_.

The following ligands and antibodies were used: human FLAG-tagged TWEAK and human IFNγ were from Enzo Life Sciences (Villeurbanne, France); mouse neutralizing anti-human TNFα (BC7) and anti-human TRAIL (B-T24) were from Diaclone (Besançon, France); the general inhibitors zVAD-fmk, CA-074, and mouse mAb anti-FLAG antibody M2 were from Sigma-Aldrich (St. Quentin Fallavier, France). PE-conjugated goat F(ab′)2 anti-mouse IgG antibody from Beckman Coulter was used for the FN14 cell surface expression experiment, and FITC-conjugated mouse mAb (ITEM-4) anti-human FN14 from Santa Cruz Biotechnology (Covalab, Paris, France) and FITC-conjugated mouse IgG2b were used as isotype controls. Rabbit polyclonal anti-human AIF (1/100) (CST, Ozyme) was used and was visualized using a FITC-conjugated goat anti-rabbit F(ab)′_2_ (1/50) antibody (Immunotech, Marseille, France). All antibodies were tested with known positive and negative control samples before use.

### Cell viability assay

HaCaT cells (5×10^3^ cells per well) and NHK (10.10^3^ cells per well) were incubated with increasing amounts (10–100 ng/ml) of TWEAK in the presence or absence of IFNγ (10 ng/ml) for 3–4 days in flat-bottom 96-well microtiter plates. Cell viability was determined by measuring the metabolic activity using an MTT (3-(4, 5-dimethylthiazol-2-yl)-2,5-diphenyltetrazolium bromide) assay. Briefly, 20 µl of a 5 mg/ml MTT solution was added to each well. Following a 4 h incubation at 37°C, the cells were lysed by the addition of 100 µl of lysis buffer (20% SDS and 50% DMF) and were incubated O/N at 37°C. Following the measurement of the optical density at 590 nm, the results were analyzed. The assays were performed in triplicate.

For the inhibition experiments, the cells were mixed with 1–10 µM zVAD-fmk and CA-074 for 10 minutes prior to dilution into the growth medium to yield a final concentration of between 1 and 10 µM during the growth phase. Stock solutions (10 mM) of zVAD-fmk and CA-074 inhibitors were in DMSO, and corresponding DMSO concentrations were run as controls in all experiments.

HaCaT cells were pre-treated with 100 ng/ml of neutralizing anti-TNFα and/or anti-TRAIL monoclonal antibodies, before the addition of the different cytokines.

### Apoptosis

After treatment, keratinocytes were trypsinized, were combined with floating cells and were then counted. The cells (2.10^4^) were suspended in annexinV binding buffer and were stained with 5 µl annexinV-FITC (Bender Medsystems, Vienna, Austria) and 10 µl propidium iodide (PI) for 10 min at room temperature in the dark. The cells were analyzed immediately using a Coulter EPICS XL flow cytometer and the data were analyzed using Win MDI software. Apoptotic cells were identified as annexinV^+^/PI^−^ (early apoptotic cells) and annexinV^+^/PI^+^ (late apoptotic cells). Each experiment was repeated at least three times.

Apoptosis was also quantified by the direct visualization of the cells under 10× magnification, and the apoptotic cells were counted and identified by the presence of characteristic membrane blebbing and nuclear chromatin condensation. Additionally, the indirect TUNEL enzymatic labeling technique was used to detect apoptotic cells using an *in situ* cell death detection kit with alkaline phosphatase (AP) (Boehringer Mannheim, Germany).

A human apoptosis array kit (R&D Systems, Lille, France) was used to measure the level of expression of pro- and anti-apoptotic proteins before and after the addition of TWEAK.

### Receptor-ligand binding assay

After detachment, the cells (1.10^6^) were incubated in cell culture medium containing 10% normal human AB serum for 30 min at 4°C. FLAG-TWEAK was added to a final concentration of 100 ng/ml. FACS buffer (1 mM EDTA, 1% BSA and 0.2% NaN3 in PBS) was used as a mock control. The binding of TWEAK was detected by the sequential addition of monoclonal mouse anti-FLAG M2 antibody and PE-conjugated goat F(ab′)2 anti-mouse IgG antibody (1/50). Fluorescence was analyzed using a Beckman-Coulter Cytomics FC 500 cytometer and WinMDI software to detect the binding of FLAG-TWEAK and fluorescent antibodies on the surface of the cells.

### Cell cycle analysis

NHK cells were treated with TWEAK (100 ng/ml) or TWEAK and EGF (50 ng/ml) for 24 h, were harvested, were washed in PBS-EDTA and were incubated for at least 2 hours at 4°C in a 30 mM sodium citrate buffer, pH 7.6, containing 0.1% Nonidet P-40, 50 µg/ml RNAse A and 50 µg/ml propidium iodide. The cells were assayed using flow cytometry in a Becton-Dickinson FACSCanto II cytometer and the data were analyzed using ModFit LT (Verify Software, Topsham, MN) software. The experiments were repeated three times. Untreated cells were used as controls.

### ELISA

HaCaT cells (500×10^3^/well) and NHK (1×10^6^) were incubated with TWEAK (100 ng/ml) for 3 (HaCaT) or 2 (NHK) days in 6-well flat bottom plates. The amount of TNFα and TRAIL secreted into the supernatants was measured using the corresponding ELISA kits (Elipair, Diaclone), according to the manufacturer's instructions.

### Quantitative RT-PCR

Total RNA was isolated from cell cultures using a miniprep RNA isolation kit (Qiagen, Hilden, Germany). RNA (1 µg) was subjected to the Thermoscript RT-PCR (Invitrogen) assay. Real-time PCR using SYBR Green was performed using a Power SYBR Green qPCR kit (Applied Biosystems, Warrington, UK) according to the manufacturer's instructions and a 7300 Real Time PCR System (Applied Biosystems). The primers were designed using Primer Express 3.0 software (Applied Biosystems). Primers were synthesized by Invitrogen.

TNFα forward (fwd) CCCAGGCAGTCAGATCATCTTC, reverse (rev) GGTTTGCTACAACATGGGCTACA;

TRAIL fwd CAGAGGAAGAAGCAACACATTGTC, rev GCGGCCCAGAGCCTTT;

LTα fwd GCCTTGGTTCTCCCCATGA, rev GTGTAGGGTGGTGCCACACA;

GAPDH fwd TGGGTGTGAACCATGAGAAGTATG, rev GGTGCAGGAGGCATTGCT;

EDA A1 fwd GCCAAGGGTCAGCAATTCA, rev TGATGCGAGACCAGTCATTGA;

FASLG fwd CCCATTTAACAGGCAAGTCCAA, rev GCAGGACAATTCCATAGGTGTCT; 14-3-3σ fwd CAAAGACAGCACCCTCATCATG, rev TCGGCCGTCCACAGTGT; Bim1 fwd GGCTGCCAGACAAGGTCAA, rev TTTATTCAGAGCTGGAGCAACAAG; GADD45A fwd TTGCAATATGACTTTGGAGGAATTC, rev CCCCCACCTTATCCATCCTT; FOXO3 fwd ACGGCTCACTCTGTCCCAGAT, rev TGTCGCCCTTATCCTTGAAGTAG.

The reaction conditions for real-time PCR were 95°C for 10 min, followed by 40 cycles of 95°C for 15 sec and 60°C for 60 sec. Changes in expression levels were normalized to control GAPDH RNA levels. Each set of primers was tested with at least three different RNA samples that were treated independently.

### Tissue selection

Specimens of normal human skin (*n* = 20), basal (*n* = 10) and squamous cell carcinoma (*n* = 10) and psoriasis (*n* = 9) were obtained from the University of Crete, School of Medicine, Department of Pathology Tissue Bank, and permission was obtained from the University Hospital Research and Ethics Committee. Slides were prepared with serial sections of tissue (3 µm): one sample for hematoxylin-eosin staining and two for the specific immunostaining of TWEAK and FN14. The slides were reviewed blindly by two independent investigators who came to a consensus.

### Immunohistochemistry

After deparaffinization and hydration, the slides (in citrate buffer; 0.01 M, pH 6.0) were subjected to three cycles (5 min each) of incubation in a microwave oven (500 W) and were treated with EDTA buffer (15 min). The specific antibodies used were TWEAK (sc-12405 goat polyclonal antibody, 1/100 dilution) and Fn14 (mouse monoclonal, sc-56250, dilution 1/50, both from Santa Cruz Biotechnology, CA, USA). A K-1500 CSA kit (DAKO, Glostrup, Denmark) was used for the immunodetection of TWEAK and Fn14. Counterstaining was performed using Mayer's hematoxylin. Known positive and negative controls (omission of the primary antibody) were used in each experiment , as previously described [45].

### Measurement of the mitochondrial membrane potential, total ROS, superoxide and oxidized glutathione levels

The mitochondrial membrane integrity was evaluated using a rhodamine 123 (R123) stain. This cationic fluorescent dye concentrates in the membrane of functional mitochondria because of the high negative electrical potential across the mitochondrial inner membrane [46], [47], which is proportional to the mitochondrial membrane potential (ΔΨm). After a 3-hour treatment with 0.02 mM, 0.2 mM or 2 mM H_2_O_2_, the cells were detached from the culture flasks and were diluted in PBS (1×10^6^ cells/ml). The cells were incubated in R123 at 1 µM for 15 min at room temperature and were assayed using flow cytometry and a Beckton-Dickinson FACSArray apparatus (Becton-Dickinson, Franklin Lakes, NJ). The data were analyzed using CELLQuest (Beckton-Dickinson) software.

Total ROS (reactive oxygen species) and superoxides were measured using a Total ROS/Superoxide detection kit (Enzo Life Sciences).

Oxidized glutathione levels were measured as previously described [48].

### Western blot analysis

The Western blots were prepared and incubated as previously described [26], were visualized using chemiluminescent reagents (Thermo Fischer Scientific, Brebières, France) and were quantified using an ImageQuant LAS 4000 apparatus (GE Health Care, Orsay, France). All primary antibodies were used at a dilution of 1/1000 and were purchased from CST, Ozyme (St Quentin en Yvelines, France), except for mcl-1, which was purchased from Abcam (Cambridge, UK). The antibodies used were mouse anti-human cyclinB1 mAb, rabbit anti-human phospho cdc2 mAb, mouse anti-human cdc2 mAb, and rabbit anti-human AIF polyclonal antibody. Secondary HRP-conjugated anti-mouse IgG (1/1000) and anti-rabbit IgG (1/2000) were used to visualize the proteins (Jackson ImmunoResearch, Baltimore, PA, USA).

### Confocal microscopy

The cells were fixed on glass slides as previously described. Images were acquired by confocal microscopy on a Zeiss LSM 510 META confocal laser microscope (Zeiss, Oberkochen, Germany) with a Plan Apochromat 63× N.A.1.4 oil-immersion objective using LSM510 software v4.0 (Zeiss). To restore image quality, the images were processed using AutoDeblur 2D deconvolution software (AutoQuantr, Watervliet, NY), which uses blind iterative algorithms.

### Data analysis

The results are expressed as the mean ± SD, and the statistical analysis was performed using the Student's *t*-test. Differences were considered significant when *P*<0.05.
